# Dating phototrophic microbial lineages with reticulate gene histories

**DOI:** 10.1111/gbi.12273

**Published:** 2018-01-31

**Authors:** C. Magnabosco, K. R. Moore, J. M. Wolfe, G. P. Fournier

**Affiliations:** ^1^ Flatiron Institute Center for Computational Biology Simons Foundation New York, NY USA; ^2^ Department of Earth, Atmospheric and Planetary Sciences Massachusetts Institute of Technology Cambridge MA USA

**Keywords:** horizontal gene transfer, molecular clock, phototrophy

## Abstract

Phototrophic bacteria are among the most biogeochemically significant organisms on Earth and are physiologically related through the use of reaction centers to collect photons for energy metabolism. However, the major phototrophic lineages are not closely related to one another in bacterial phylogeny, and the origins of their respective photosynthetic machinery remain obscured by time and low sequence similarity. To better understand the co‐evolution of Cyanobacteria and other ancient anoxygenic phototrophic lineages with respect to geologic time, we designed and implemented a variety of molecular clocks that use horizontal gene transfer (HGT) as additional, relative constraints. These HGT constraints improve the precision of phototroph divergence date estimates and indicate that stem green non‐sulfur bacteria are likely the oldest phototrophic lineage. Concurrently, crown Cyanobacteria age estimates ranged from 2.2 Ga to 2.7 Ga, with stem Cyanobacteria diverging ~2.8 Ga. These estimates provide a several hundred Ma window for oxygenic photosynthesis to evolve prior to the Great Oxidation Event (GOE) ~2.3 Ga. In all models, crown green sulfur bacteria diversify after the loss of the banded iron formations from the sedimentary record (~1.8 Ga) and may indicate the expansion of the lineage into a new ecological niche following the GOE. Our date estimates also provide a timeline to investigate the temporal feasibility of different photosystem HGT events between phototrophic lineages. Using this approach, we infer that stem Cyanobacteria are unlikely to be the recipient of an HGT of photosystem I proteins from green sulfur bacteria but could still have been either the HGT donor or the recipient of photosystem II proteins with green non‐sulfur bacteria, prior to the GOE. Together, these results indicate that HGT‐constrained molecular clocks are useful tools for the evaluation of various geological and evolutionary hypotheses, using the evolutionary histories of both genes and organismal lineages.

## 1 INTRODUCTION

The ability to acquire energy through photon capture, or phototrophy, is found within seven major lineages of the bacterial domain (Cardona, 2015). Despite this broad taxonomic distribution, the photosynthetic reaction centers (RCs) responsible for harnessing and converting photons to energy are believed to have evolved from a single common ancestor (Blankenship, 2010). These photosynthetic RCs are divided into two classes: FeS‐type (Type I) and Quinone‐type (Type II). All anoxygenic phototrophs use either FeS‐Type or Quinone‐Type RCs; however, Cyanobacteria use both FeS‐Type and Quinone‐Type RCs, along with an oxygen‐evolving complex, to strip electrons from water and produce O_2_ (Hohmann‐Marriott & Blankenship, 2011). The ability to perform oxygenic photosynthesis and the origins of the coexistence of both types of RCs in Cyanobacteria are long‐standing questions in the study of the evolution of phototrophs (for a review, see Hohmann‐Marriott and Blankenship (2011)).

Multiple hypotheses have been proposed to account for the coexistence of both types of RC's in Cyanobacteria, including fusion (Blankenship, 1992; Mathis, 1990), selective loss (Olson, 1970; Olson & Pierson, 1987a,b), and duplication (Allen & Martin, 2007). The fusion hypothesis proposes that the FeS‐Type and Quinone‐Type RCs developed within different lineages, and the ancestor lineage of Cyanobacteria acquired one or both of the RCs through horizontal gene transfer (HGT). The selective‐loss hypothesis proposes that an ancestral photosynthetic organism evolved both FeS‐Type and Quinone‐Type RCs and either the FeS‐Type or Quinone‐Type RC was lost in the respective ancestor of anoxygenic phototroph lineages. The duplication hypothesis proposes that a “protocyanobacterium” gave rise to the FeS‐Type and Quinone‐Type RCs through a gene duplication event and subsequently transferred the RCs to other lineages through HGT.

All three hypotheses necessitate the transfer of RCs. Previous work has attempted to constrain the directionality of these transfers via inferences from the complex evolutionary history of chlorophyll biosynthesis genes (Sousa, Shavit‐Grievink, Allen, & Martin, 2013) supporting reaction center duplication, rather than fusion, early in the cyanobacterial stem lineage, and by a detailed analysis of reaction center subunit evolution, including molecular clock estimates on the divergence times for these protein families (Cardona, Sanchez‐Baracaldo, Rutherford, & Larkum, 2017) supporting selective loss of photosystems rather than photosystem merger within stem Cyanobacteria. Each of these approaches draws inferences from mapping the evolutionary history of specific components of the photosynthetic machinery. However, any such hypothesis must also be consistent with the history and timing of the organismal lineages that presumably donated and/or acquired these genes and phenotypes.

As FeS‐Type and Quinone‐Type RCs are believed to pre‐date the rise and expansion of the phototrophic lineages (Cardona, 2015), the ancestor of each phototrophic clade likely possessed an evolved FeS‐Type, Quinone‐Type, or (in the case of Cyanobacteria) FeS‐Type and Quinone‐Type RC. This also constrains early RC HGTs to have occurred between the stem groups of the phototrophic clades and requires the stem lineage of HGT donors to pre‐date the crown groups of HGT recipients. Using molecular clocks, we are able to better explore these RC HGT scenarios in the context of (arguably) the three most ancient phototrophic groups, Cyanobacteria (FeS‐Type and Quinone‐Type RCs) (Allen & Martin, 2007), green sulfur bacteria (GSB; FeS‐Type RC) (Cavalier‐Smith, 2001), and green non‐sulfur bacteria (GNS; Quinone‐Type RC) (Woese, 1987). Other phototrophic groups are excluded from this analysis as they occupy derived positions within bacterial phylogenies. These lineages are likely to be more recent recipients of photosynthetic machinery and include the polyphyletic purple bacteria distributed throughout the Alphaproteobacteria, Betaproteobacteria, and Gammaproteobacteria classes (Nagashima & Nagashima, 2013) and Heliobacteria within Clostridia (Sattley & Swingley, 2013). Therefore, we do not consider additional hypotheses in which these lineages could be donors of photosystems to the cyanobacterial stem lineage. Analysis of the posterior age distributions allows us to evaluate the feasibility of each RC HGT in and out of stem Cyanobacteria as well as infer the geological context in which these phototrophic taxa evolved.

In recent years, several date estimates for the origins of Cyanobacteria (Blank & Sanchez‐Baracaldo, 2010; Dvořák et al., 2014; Falcón, Magallón, & Castillo, 2010; Sánchez‐Baracaldo, 2015; Schirrmeister, de Vos, Antonelli, & Bagheri, 2013; Schirrmeister, Gugger, & Donoghue, 2015; Shih, Hemp, Ward, Matzke, & Fischer, 2017) and for the Quinone‐Type RCs of photosystem II (Cardona et al., 2017) have been obtained using molecular clock analyses. For Cyanobacteria, date estimates vary by up to 1 Ga due to the strong influence of the fossil calibrations and priors used in such analyses (Schirrmeister, Sanchez‐Baracaldo, & Wacey, 2016). The recent discovery of two related, non‐photosynthetic outgroups to Cyanobacteria, Melainabacteria (Di Rienzi et al., 2013), and Sericytochromatia (Soo, Hemp, Parks, Fischer, & Hugenholtz, 2017) (formerly *ML635J‐*21), has greatly improved our ability to infer the evolutionary history of oxygenic photosynthesis and RCs. The absence of photosynthetic machinery in either of these outgroups is parsimonious with oxygenic photosynthesis evolving within the Cyanobacteria/Melainabacteria/Sericytochromatia (CMS) Group after the divergence of Melainabacteria, either via a fusion event via HGT of photosystems from other groups (Soo et al., 2017), or photosystem origin and duplication within this lineage itself (Allen & Martin, 2007). Due to the expansion of this lineage, the renaming of O_2_‐producing Cyanobacteria to “Oxyphotobacteria” has been proposed to reflect the derived character of oxygenic photosynthesis acquired in the ancestor lineage of this group (Shih et al., 2017; Soo et al., 2017). By including the non‐photosynthetic lineages of the CMS Group, we are able to shorten the Cyanobacteria stem lineage along which RC HGT likely occurred and increase our ability to discriminate between specific HGT hypotheses.

It is now well understood that processes (like HGT) generate conflicts between individual gene histories. These HGTs can be used as an informative character for the evolutionary history of organismal lineages (Bapteste et al., 2009; Huang & Gogarten, 2009; Soucy, Huang, & Gogarten, 2015). Traditionally, molecular clock methods model the evolutionary history of a genome within a microbial lineage as a strictly bifurcating tree; however, HGTs can also provide relative constraints between nodes (e.g., A>B) as they establish the relative timing of donor and recipient lineages. Previous analyses have incorporated this temporal dimension in the simultaneous reconstruction of supertree phylogenies and relative divergence times from individual gene trees, including reticulating branches (Szöllősi, Boussau, Abby, Tannier, & Daubin, 2012). When applied to a fixed species tree, HGT events can also propagate absolute temporal constraints under certain criteria: (i) The topology of the transfer event must be sufficiently resolved, with a well‐supported branch within the ancestor of the donor lineage; (ii) the phylogeny of the donor and recipient clades must be sufficiently similar to their respective species trees, ensuring that the reticulation is not complicated by multiple parallel or subsequent HGT events; and (iii) there must be an absolute date calibration associated with either the donor or (ideally) the recipient lineage crown group (Wolfe & Fournier, 2017). We refer to HGTs that meet these criteria as “index HGTs,” roughly analogous to the concept of “index fossils” from traditional paleontological biostratigraphy. Using this approach, to model the evolution of phototrophs, we are able to better constrain the direction of RC HGTs as well as inform the relative ages of the crown and stem groups of the participant clades.

## 2 MATERIALS AND METHODS

### 2.1 Species tree reconstruction

Ninety‐five bacterial genomes (Table S1) were collected from the NCBI (http://www.ncbi.nlm.nih.gov/) and PATRIC (https://www.patricbrc.org/) databases based on their ability to represent the phototrophic (Cyanobacteria, GNS, and GSB) and outgroup (Sericytochromatia, Melainabacteria, Chloroflexi, Alphaproteobacteria, Ignavibacteria, and Bacteroidetes) taxa selected for this study. BLASTp was used to search each genome for homologous genes of interest (Table S2). A concatenated alignment (Data S1) of 30 ribosomal proteins (Table S2) was generated using muscle v3.8.31 (Edgar, 2004). raxml v8.1.9 (‐m protgammalg4m) (Stamatakis, 2014) was used to reconstruct the species tree.

In instances where a random or neighbor‐joining start tree was used for the estimation of a maximum‐likelihood (ML) phylogeny, the best scoring ML tree was inconsistent with previous reports on cyanobacterial phylogeny (Data S2) (Sericytochromatia and Melainabacteria were placed sister to each other rather than the previously reported topology of Sericytochromatia placed basal to Melainabacteria and Cyanobacteria). Sericytochromatia, however, was identified as basal to Melainabacteria and Cyanobacteria in 10 of the 100 bootstrap trees. Therefore, a guide tree (Data S3) was used to force Sericytochromatia basal to the Melainabacteria/Cyanobacteria split and generate a phylogeny (Figure S1; Data S4) in agreement with the 16S SSU ribosomal RNA phylogeny (Data S5) that places Sericytochromatia as the most basal CMS Group lineage. This placement is further supported by the HGT of the gene encoding SahH from within Chloroflexi to the common ancestor of Melainabacteria and Cyanobacteria, excluding Sericytochromatia, as described in the methods section *Horizontal Gene Transfer Constraints* below. Further discussion on this topic and age distributions estimated under the best scoring ML phylogeny (Table S3) are provided in the supplement.

### 2.2 Divergence time estimation

Divergence time analyses were performed using phylobayes v3.3 (−catfix C20 −ugam −nchain 2 100 0.3 50, all other parameters default) (Lartillot, Lepage, & Blanquart, 2009). A description of the models used in this analysis is provided in Table 1. Chronograms were generated using the “readdiv” command of phylobayes. For each model, the first N × 0.2 saved points (N) were excluded the computation of the chronogram. The 95% highest posterior density (HPD) intervals were calculated from the “datedist” file (Data S6). Posterior date estimates were compared to those obtained under the prior using the “‐prior” function in phylobayes (Supporting Discussion).

**Table 1:** Model Summary. When applicable, models were run using either a 1.2 Ga or 1.6 Ga akinete constraint along with the calibrations indicated in Figure 1 and Table 2

Cyanobacteria models	Root prior		Taxa included	Akinete calibration?	Great Oxidation Event calibration?
	Age	Shape			
*Gloeobacter* Outgroup 1 (Schirrmeister et al., 2013, 2015)	3,800 to 2,450 Ma	Flat	Cyanobacteria	Yes	Yes (root)
*Gloeobacter* Outgroup 2 (Blank & Sanchez‐Baracaldo, 2010)	2,700 to 2,320 Ma	Flat	Cyanobacteria	Yes	Yes (root)
*Gloeobacter* Outgroup 3 (Sánchez‐Baracaldo, 2015)	2,500 ± 200 Ma	Normal	Cyanobacteria	Yes	No
Alphaproteobacteria Outgroup 1 (Shih et al., 2017)	3,800 to 2,400 Ma	Flat	Cyanobacteria, Melainabacteria, Alphaproteobacteria	Yes	Yes (crown Cyanobacteria)
Alphaproteobacteria Outgroup 2 (Shih et al., 2017)	3,800 to 2,400 Ma	Flat	Cyanobacteria, Melainabacteria, Alphaproteobacteria	Yes	Yes (stem Cyanobacteria)
Alphaproteobacteria Outgroup 3 (Shih et al., 2017)	3,800 to 2,400 Ma	Flat	Cyanobacteria, Melainabacteria, Alphaproteobacteria	Yes	No
Alphaproteobacteria Outgroup 4 (Shih et al., 2017)	3,800 to 2,400 Ma	Flat	Cyanobacteria, Melainabacteria, Alphaproteobacteria	No	No

Phototroph models	Root prior		Taxa included	SahH HGT?	BchH HGT?
	Age	Shape			
(A) Phototroph Tree	3,900 ± 200 Ma	Normal	All	No	No
(B) SahH	3,900 ± 200 Ma	Normal	All	Yes	No
(C) BchH	3,900 ± 200 Ma	Normal	All	No	Yes
(D) SahH+BchH	3,900 ± 200 Ma	Normal	All	Yes	Yes

**Figure 1:**
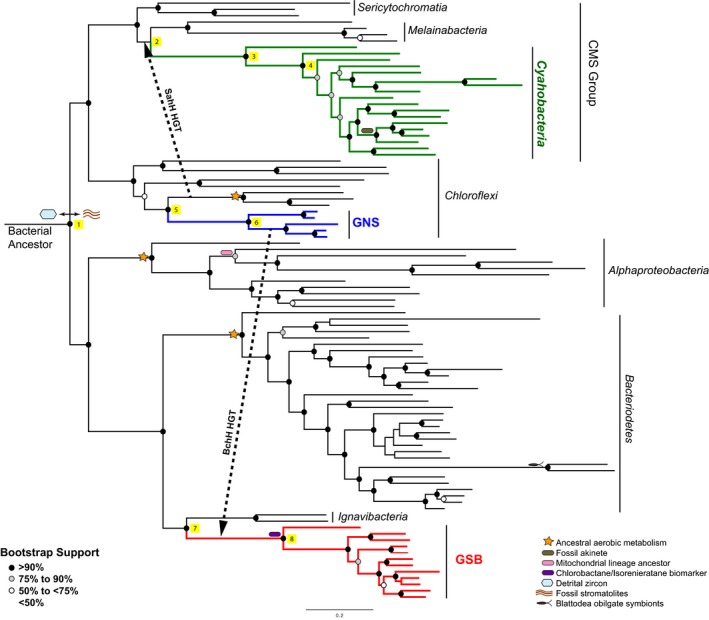
Phylogenetic relationship of taxa included in this study. A phylogenetic tree derived from the alignment of 30 ribosomal proteins is displayed. Cyanobacteria (green), GNS (blue), and GSB (red), and major groups of taxa are labeled. Individual tips of the same phylogenetic tree are labeled inFigure S1. Illustrations of the calibrations used in the molecular clock analyses are placed to the left of the node that they constrain. Further description of these calibrations can be found in the legend and Table 2. Circles on the nodes indicate bootstrap values that are further described in the legend. Yellow squares 1–8 are placed to the right of nodes and are further described in Table 3

In an effort to compare our results to previously published literature, we designed two Cyanobacteria‐centric models. These models are our “*Gloeobacter* Outgroup” Model and “Alphaproteobacteria Outgroup” Model (Table 1). As the name suggests, the *Gloeobacter* Outgroup Model does not include a deep bacterial outgroup with a root corresponding to the last bacterial common ancestor (LBCA). Instead, *Gloeobacter violaceus* PCC 7421 is the outgroup to other cyanobacterial taxa (Data S7). The “Alphaproteobacteria Outgroup” Model was designed to reflect the analyses of Shih et al. (2017). Here, a phylogenetic tree consisting of Cyanobacteria, Melainabacteria, and Alphaproteobacteria (Data S8) was calibrated with the mitochondrial constraint (Figure 1), the “Rise of Oxygen” constraints proposed by Shih et al., and a flat root prior of 3.8 Ga to 2.4 Ga. With the exception of Alphaproteobacteria Outgroup Model 4, a 1.2 Ga or 1.6 Ga akinete constraint was used as an internal constraint for all models.

### 2.3 Date constraints

We constrain the timing of the last common bacterial ancestor (LCBA) by the earliest evidence of habitability (4.4 Ga zircons) (Wilde, Valley, Peck, & Graham, 2001) and the earliest direct evidence of diverse bacterial communities (3.5 Ga stromatolites in the Warrawoona Group of Australia) (Schopf & Packer, 1987). While the latter may represent phototrophic metabolisms and lineages themselves, their taxonomic affinities are unknown and thus are not suitable for use as a direct calibration on groups within our analysis. This constraint was applied via a normally distributed gamma root prior (3900 Ma, SD 200 Ma). We selected this distribution for our analyses due to our decreasing belief that the LCBA (the root of our phylogenetic tree) originated near the 4.4 Ga and 3.5 Ga historical time points. Summaries of the internal constraints are provided in Table 2. These calibrations include constraints derived from aerobic respiration metabolisms postdating the rise of oxygen, the mitochondrial ancestor in Alphaproteobacteria (Gray, Burger, & Lang, 1999) pre‐dating the cytoskeletal‐containing protistan fossils of the Roper Group (Australia) (Javaux, Knoll, & Walter, 2001), Blattodea obligate symbionts in Bacteroidetes (Lo, Bandi, Watanabe, Nalepa, & Beninati, 2003; Wolfe, Daley, Legg, & Edgecombe, 2016), isorenieratane and chlorobactane biomarkers identified in the 1.64 Ga Barney Creek Formation (Brocks & Schaeffer, 2008), and fossil akinetes (rod‐like resting cells) interpreted as cyanobacterial fossils (Horodyski & Donaldson, 1980; Sharma, 2006). As the age of fossil akinetes has been contested, all models were run with either a −1 to 1.2 Ga akinete calibration [*Archaeoellipsoides* fossils from the Dismal Lakes Group of Arctic Canada (Horodyski & Donaldson, 1980)] or −1 to 1.6 Ga akinete calibration (Semri Group of India) (Sharma, 2006). All calibration files are included in Data S9.

**Table 2:** PhyloBayes calibration file inputs. This table provides the upper and lower limits of calibrations identified calibration file of PhyloBayes runs. The nodes on which these constraints are applied are identified in Figure 1

Date constraint	Upper limit (Ma)	Lower limit (Ma)
Ancestral aerobic metabolism	2,450	−1
Fossil akinete	−1	1,200 or 1,600
Mitochondrial lineage ancestor	−1	1,400
Chlorobactane/Isorenieratane biomarker	−1	1,640
Blattodea obligate symbiont	411	125.71

### 2.4 Horizontal gene transfer constraints

For this study, we identified two index HGTs between the taxonomic groups surveyed. The first index HGT used was a transfer of a gene encoding Mg‐chelatase (BchH) from within GNS to stem GSB (Data S10) that was originally identified by Sousa et al. (2013). This gene also underwent a clear duplication in the GSB stem lineage subsequent to transfer. We also identified a second index HGT of a gene encoding S‐adenosyl‐L‐homocysteine hydrolase (SahH) from within Chloroflexi to the common ancestor of Cyanobacteria and Melainabacteria (Data S11). HGT information was incorporated into our models by post‐sampling the posterior for steps in MCMC chains where the HGT conditional constraints were met (Data S6), permitting multiple HGT constraints to be included in a single analysis.

## 3 RESULTS AND DISCUSSION

### 3.1 Divergence date estimates for phototrophic taxa

The results of Phototroph Models A and D (see Table 1 for description) are shown in Table 3. The results of Models B and C are included in the Table S4. Under the 1.2 Ga akinete‐constrained Phototroph Model A, Cyanobacteria are estimated to be the oldest phototrophic lineage with a median stem age of 2825 Ma (95% HPD = 2570 Ma to 3195 Ma) and median crown age of 2305 Ma (95% HPD = 2026 Ma to 2601 Ma). GNS are estimated to be the youngest group (median crown age = 1041 Ma) with the largest amount of uncertainty in their crown age estimate (95% HPD = 624 Ma to 1542 Ma). When Model A's 1.2 Ga akinete constraint is replaced by a 1.6 Ga akinete constraint, Cyanobacteria stem and crown divergence date estimates are shifted ~350 Ma older, while crown GNS and GSB date estimates are relatively unchanged (Table 3).

**Table 3:** Divergence date estimates. Median divergence date estimates are given in Ma. () denotes the 95% HPD calculated from the *datedist output of PhyloBayes

	Node ID	1.2 Ga Akinete constraint		1.6 Ga Akinete constraint	
		Model A	Model D	Model A	Model D
Rootage	1	3,591 Ma (3,323–3,837 Ma)	3,624 Ma (3,483–3,884 Ma)	3,692 Ma (3,408–3,968 Ma)	3,692 Ma (3,569–3,920 Ma)
Stem Cyanobacteria	2	2,825 Ma (2,570–3,195 Ma)	2,776 Ma (2,396–2,934 Ma)	3,158 Ma (2,886–3,486 Ma)	2,836 Ma (2,724–2,936 Ma)
Crown Cyanobacteria	3	2,305 Ma (2,026–2,601 Ma)	2,244 Ma (1,912–2,419 Ma)	2,677 Ma (2,426–2,972 Ma)	2,515 Ma (2,382–2,593 Ma)
Crown Cyanobacteria excluding *Gloeobacter*	4	1,943 Ma (1,723–2,188 Ma)	1,902 Ma (1,713–2,096 Ma)	2,328 Ma (2,098–2,564 Ma)	2,234 Ma (2,087–2,360 Ma)
Stem GNS	5	2,099 Ma (1,584–2,674 Ma)	2,861 Ma (2,498–3,004 Ma)	2,253 Ma (1,597–2,785 Ma)	2,922 Ma (2,759–2,950 Ma)
Crown GNS	6	1,041 Ma (624–1,542 Ma)	1,986 Ma (1,682–2,433 Ma)	1,111 Ma (618–1,714 Ma)	2,109 Ma (1,810–2,438 Ma)
Stem GSB	7	2,640 Ma (2,298–2,977 Ma)	2,561 Ma (2,263–2,853 Ma)	2,602 Ma (2,265–2,981 Ma)	2,737 Ma (2,423–2,902 Ma)
Crown GSB	8	1,798 Ma (1,641–2,112 Ma)	1,716 Ma (1,645–1,950 Ma)	1,793 Ma (1,640–2,112 Ma)	1,719 Ma (1,660–2,173 Ma)

When the SahH and BchH HGT constraints are applied (Phototroph Model D), GNS and Cyanobacteria are the oldest phototrophic lineages, and date estimate precision is improved. In particular, the 95% HPD intervals of stem GNS divergence date estimates are reduced by >50% of the non‐HGT‐constrained HPD interval. When only the SahH or BchH HGT constraint is applied (Models B and C), the uncertainty in the age estimate of GNS is restored (Table S4). The sensitivity of the GNS lineage to the HGT constraints suggests that the GNS are a slow‐evolving lineage that are difficult to estimate using molecular dating methods and may require additional calibrations for firm age estimates to be made.

### 3.2 Origin of phototrophs and their relationship to geologic time

The evolutionary history of phototrophy is innately tied to the biochemical history of our planet. It is generally accepted that the loss of the mass‐independent sulfur isotope fractionations from the sedimentary record ~2.4 to ~2.3 Ga (Bekker et al., 2004; Farquhar, Bao, & Thiemens, 2000; Luo et al., 2016) marks the transitional period prior to the stabilization of an oxygenated atmosphere ~10^−5^ times the present atmospheric O_2_ level. Although commonly referred to as the Great Oxidation Event (GOE), this transition was not a discrete event signifying at the onset of O_2_ production. Various forms of geological (e.g., banded iron formations and stromatolites) and geochemical (e.g., sterane biomarkers, trace metal enrichments, and organic‐rich shales) features in the rock record have been reported as evidence for oxygen production prior to the GOE [For a comprehensive review, see: (Lyons, Reinhard, & Planavsky, 2014)]. Although abiogenic sources of O_2_ have been reported for the Archaean (Gaillard, Scaillet, & Arndt, 2011; Kump & Barley, 2007), Cyanobacteria performing oxygenic photosynthesis are generally accepted to be the main contributor of O_2_ prior to and throughout the GOE.

**Figure 2:**
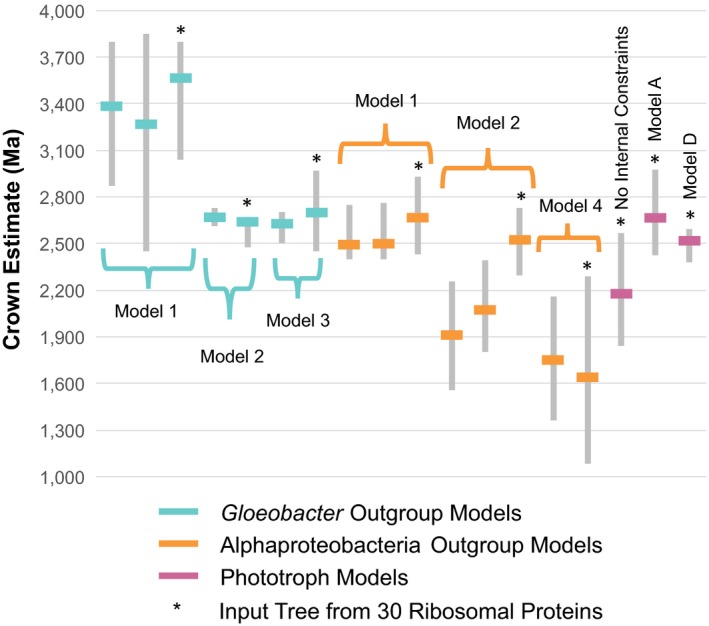
Comparison of cyanobacteria crown divergence date estimates under different models. Cyanobacteria crown date estimates for *Gloeobacter* Outgroup (cyan), Alphaproteobacteria Outgroup (orange), and Phototroph Models (pink) using a 1.6 Ga akinete constraint (where applicable) are shown. Gray bars represent the 95% HPD, and the solid lines indicate the median of the Cyanobacteria crown date estimates. Date estimates derived from this study (phylogenetic tree from 30 ribosomal proteins) are indicated by a *. These estimates vary from other published models (bars without stars) using similar constraints but different input phylogenies (e.g., 16S SSU ribosomal RNA). From left to right, the molecular clock results used to derive the date estimates are as follows: Schirrmeister et al. (2013, 2015), *Gloeobacter* Outgroup Model 1, Blank and Sanchez‐Baracaldo (2010), *Gloeobacter* Outgroup Model 2, Sánchez‐Baracaldo (2015), *Gloeobacter* Outgroup Model 3, T72 (Shih et al., 2017), T73 (Shih et al., 2017), Alphaproteobacteria Outgroup Model 1, T64 (Shih et al., 2017), T65 (Shih et al., 2017), Alphaproteobacteria Outgroup Model 2, T69 (no constraints within Cyanobacteria) (Shih et al., 2017), Alphaproteobacteria Outgroup Model 4, Phototroph Model without internal constraints, Phototroph Model A, Phototroph Model D

Bayesian molecular dating is a widely used method to study the evolution and diversification of Cyanobacteria in relation to the GOE. Although several molecular clocks have been generated for Cyanobacteria (Dvořák et al., 2014; Falcón et al., 2010; Sánchez‐Baracaldo, 2015; Schirrmeister et al., 2013, 2015; Shih et al., 2017), large differences in outgroup, root prior, and internal calibration selection in these models have resulted in a wide variety of date estimates for the origin of oxygenic phototrophs*—*ranging from younger than 2.0 Ga (Shih et al., 2017) to older than 3.4 Ga (Schirrmeister et al., 2013). To compare our phototroph models to publications within the literature, we designed 3 “*Gloeobacter* Outgroup” and 4 “Alphaproteobacteria Outgroup” models with the same internal calibrations as our phototroph models (Table 1; Figure 2). Despite containing the same taxa and internal calibrations, median age estimates of crown Cyanobacteria in the *Gloeobacter* Outgroup Models ranged from 2642 Ma (1.6 Ga akinete; *Gloeobacter* Outgroup Model 2) to 3563 Ma (1.6 Ga akinete; *Gloeobacter* Outgroup Model 1). The generous root prior (3.8 Ga to 2.45 Ga) of *Gloeobacter* Outgroup Model 1 led to the least precise date estimates of Cyanobacteria taxa (95% HPD intervals as large as 1583 Ma). Although the influence of root priors on molecular clock analyses has been previously reported (Warnock, Yang, & Donoghue, 2011), the direct implications of this uncertainty on the interpretation of the diversification of Cyanobacteria are worth noting. In particular, when *Gloeobacter* Outgroup Models are generated, the root age estimate is commonly interpreted as the date at which oxygenic photosynthesis has emerged. Consequently, the GOE has been incorporated into the root prior inputs of many Cyanobacteria molecular clocks, and estimates of the “emergence of oxygenic photosynthesis” were directly constrained by how precise the selected root priors were.

Shih et al. (2017) improved upon the *Gloeobacter* Outgroup Models by including Alphaproteobacteria and the sister group to Cyanobacteria, Melainabacteria, as outgroups to Cyanobacteria (Alphaproteobacteria Outgroup Model). Under this sampling, the root of the phylogeny is interpreted as the LBCA, and the stem and crown age of Cyanobacteria can be inferred. When internal calibrations are omitted, the generous root prior (3.8 Ga to 2.45 Ga) results in very young Cyanobacteria date estimates (Alphaproteobacteria Outgroup Model 4; Figure 2). This broad of a root prior appears to be problematic as the common ancestor of Melainabacteria and Cyanobacteria postdate the GOE, a prior assumption incompatible with oxygenic photosynthesis arising after the divergence of these lineages and within the cyanobacterial stem lineage. In instances where a GOE constraint (Table 1) was applied to either the crown (Alphaproteobacteria Outgroup Models 1) or stem (Alphaproteobacteria Outgroup Models 2) of Cyanobacteria, calibrated nodes were prevented from moving younger than the GOE (Figure 2) but necessitated an a priori assumption to be made about the diversification of Cyanobacteria in accordance with the GOE. In this study, we are able to avoid this caveat by including additional outgroup taxa (Sericytochromatia, Chloroflexi, Bacteroidetes, GSB, and Ignavibacteria) and a narrower, but still broad root prior (3900 Ma, *SD* = 200 Ma). When internal calibrations are removed from our phototroph models, the crown age of Cyanobacteria is estimated to be younger than GOE (2172 Ma) but the stem age of Cyanobacteria (2892 Ma) allows sufficient time for the evolution of oxygenic photosynthesis within the Cyanobacteria stem lineage prior to the peak of the GOE 2.33 Ma (Luo et al., 2016) (Figure 2, Table S5).

To the best of our knowledge, our phototroph models provide the first divergence date estimates of Cyanobacteria independent of a direct GOE constraint. Using a 1.2 Ga akinete‐calibrated Phototroph Model D, the phototrophic cyanobacterial lineages split from non‐phototrophic lineages ~2.8 Ga (Cyanobacteria stem) and crown Cyanobacteria arise ~2.2–2.3 Ga (Table 3). In this scenario, the diversification of extant members of Cyanobacteria appears to be closely linked to the GOE, a conclusion similar to that reported by Cardona et al., 2017. The 1.6 Ga akinete‐calibrated Model D provides a slightly different scenario. Here, Cyanobacteria diverge from the non‐photosynthetic lineages ~2.8 Ga but crown Cyanobacteria emerge ~2.5 Ga, and Cyanobacteria‐containing thylakoid membranes appear ~2.2 Ga (Table 3). These age intervals suggest that an increase in photosynthetic efficiency at the GOE could be due to physiological innovation within crown Cyanobacteria, namely the evolution of the thylakoid membrane. With the exception of *Gloeobacter* (the earliest branching lineage of Cyanobacteria) whose RCs are located along the plasma membrane, cyanobacterial RCs are located within thylakoid membranes (Vothknecht & Westhoff, 2001). This difference in cellular structure limits the photosynthetic capacity of *Gloeobacter*, which is far less than its thylakoid‐containing relatives (Vothknecht & Westhoff, 2001). If the GOE traverses the branch along which thylakoid membranes evolved, photosystem compartmentalization can provide an additional biological hypothesis for the rise of atmospheric oxygen during the GOE. Date estimates for crown Cyanobacteria are sensitive to the akinete constraint (1.2 Ga or 1.6 Ga) used; however, Model D date estimates of stem Cyanobacteria using either constraint is ~2.8 Ga (Table 3). These similarly old stem Cyanobacteria date estimates are compatible with oxygenic interpretations of the Archean trace metal record, although this in itself does not provide additional evidence for these interpretations (Crowe et al., 2013).

Although the GOE marked a rise in the partial pressure of atmospheric oxygen, there is strong evidence that the deep oceans remained anoxic until the Gaskiers glaciation ~580 Ma (for a comprehensive review, see: Meyer & Kump, 2008). Rather than fully oxygenating Earth's oceans, the GOE marks a transition from a ferruginous state to a stratified ocean with the potential for sulfidic conditions (Meyer & Kump, 2008). This transition is highlighted by the loss of the banded iron formations (BIFs) from the sedimentary rock record ~1.8 Ga (Cloud, 1972; Holland, 1984). Although a more complex picture of oceanic Fe/S ratios throughout the Proterozoic is coming to light (Lyons et al., 2014), increased rates of anoxygenic photosynthesis and aerobic heterotrophy are believed to have maintained the low levels of O_2_ throughout this period (Johnston, Wolfe‐Simon, Pearson, & Knoll, 2009). In modern anoxic waters, GSB have been found to account for as much as 83% of total productivity when H_2_S concentrations are high (Culver & Brunskill, 1969; Van Gemerden & Mas, 1995) and are likely to be important members of Proterozoic marine microbial communities (Johnston et al., 2009). In all phototroph models of this study, crown GSB appear ~1.7 to 1.8 Ga—just after the loss of BIFs from the sedimentary record (Table 3). Today, almost all known GSB couple anoxygenic photosynthesis to sulfide oxidation (Van Gemerden & Mas, 1995); however, one derived GSB, *Chlorobium ferrooxidans,* has been found to oxidize Fe(II) rather than sulfide (Heising, Richter, Ludwig, & Schink, 1999). As the most basal GSB are sulfide oxidizers, our date estimates suggest that modern sulfide‐oxidizing GSB arose to fill a niche introduced by increased levels of H_2_S in the oceans. These sulfide‐oxidizing GSB are likely to have outcompeted their Fe(II)‐oxidizing relatives that lived in Fe(II)‐rich waters prior to the GOE. Today, ferruginous ocean analogs are rare; however, a stratified lake in Indonesia (Crowe et al., 2008) provides the most compelling evidence for an Fe(II)‐oxidizing GSB in an Archaean Fe ocean.

### 3.3 Direction of RC HGT

Further supporting Fe(II) or alternative modes of phototrophy are the 3.4 Ga fossilized mat structures restricted to the photic zone of the Buck Reef Chert (Barberton Greenstone Belt, South Africa) (Tice & Lowe, 2004, 2006) that pre‐date the stem ages of all phototrophic lineages estimated by our models (Table 3). If these mat structures are indeed evidence of Archaean phototrophy, the earliest phototrophs must pre‐date the stem ages of all phototrophs included in our study and suggest that: (i) An ancestral RC (RC*) pre‐dates the stem ages of all phototrophic taxa included in our models, and either (iia) the ability to perform phototrophy must have been lost within at least one of the non‐photosynthetic sister lineages (e.g., Melainabacteria, Ignavibacteria, and/or Thermomicrobia) or (iib) these structures were formed by more ancient phototrophic lineages that do not have known extant descendants. With these constraints, we can explore the possibility of RC HGT in and out of Cyanobacteria using Phototroph Model D (Table 1).

**Figure 3:**
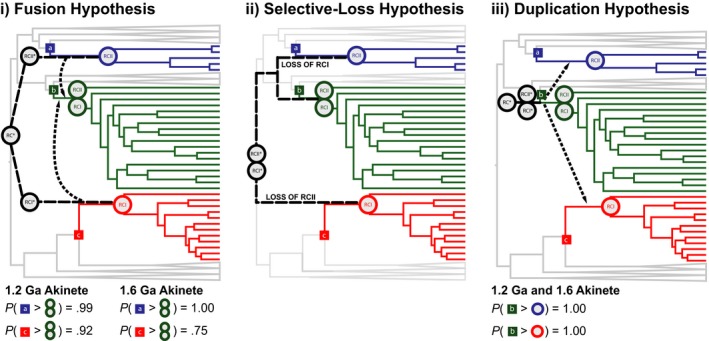
Models and probabilities of reaction center horizontal gene transfer. Illustrations of the fusion (i), selective loss (ii), and duplication (iii) hypotheses of RC evolution are provided in black. GNS (blue), Cyanobacteria (green), and GSB (red) are provided for reference in the underlying species tree. For each figure, RC* represents an ancestral RC; RCI* and RCII* represent the ancestral FeS‐Type and Quinone‐Type RCs discussed in the text. The RCs specific to each lineage are placed at the crown of each phototrophic lineage and are colored according to the schematic described above. The stems of GNS (a), Cyanobacteria (b), and GSB (c) are indicated by the colored squares. The probabilities that stem GNS is older than crown Cyanobacteria (*P*(a>green circles)) and stem GSB is older than crown Cyanobacteria (*P*(c>green circles)) are provided in (i). The probabilities that stem Cyanobacteria is older than crown GNS (*P*(b>blue circle)) and stem Cyanobacteria is older than crown GSB (*P*(b>red circle)) are provided in (iii)

The fusion hypothesis for the evolution of FeS‐Type and Quinone‐Type RCs in Cyanobacteria necessitates the divergence of FeS‐Type and Quinone‐Type RCs prior to the stem age of Cyanobacteria and the HGT of either a FeS‐Type or Quinone‐Type RCs into the Cyanobacteria stem (Figure 3a). In order for the fusion hypothesis to be valid for these taxa, the stem of the RC donor group (GNS or GSB) must be older than the crown of Cyanobacteria. We are able to calculate the probability of each of these scenarios (*P*(stem date estimate > crown date estimate)) from the posterior date estimates of Phototroph Model D (Supporting Discussion). Under this model, the HGT of a Quinone‐Type RC from GNS to Cyanobacteria is more probable (Model D, 1.2 Ga akinete: 0.99; Model D, 1.6 Ga akinete: 1.00) than the HGT of a FeS‐Type RC from GSB to Cyanobacteria (Model D, 1.2 Ga akinete: 0.92; Model D, 1.6 Ga akinete: 0.75), although there is sufficient overlap between the estimated ages of these stem lineages to permit either scenario. We also cannot exclude the possibility of an extinct HGT donor lineage for RC transfers into both GNS and Cyanobacteria.

Alternatively, both ancestral FeS‐Type and Quinone‐Type RCs may have been transferred from the stem lineage of Cyanobacteria to the anoxygenic phototroph lineages (Figure 3c). The duplication hypothesis is one such scenario and would require the transfer of a FeS‐Type or Quinone‐Type RC to GSB and GNS, respectively, from cyanobacterial stem lineages that diverged prior to crown Cyanobacteria. These transfers are highly probable (1.00) under the 1.2 Ga and 1.6 Ga akinete‐constrained Model D (Figure 3c). Under the 1.6 Ga akinete‐constrained Model D, stem and crown Cyanobacteria ages are old enough that the ancestral FeS‐Type and Quinone‐Type RCs could be transferred to anoxygenic phototrophs prior to the GOE (crown Cyanobacteria = 2515 Ma) and, thus, allow for anoxygenic photosynthesis to develop within both GNS and GSB prior to the GOE. A pre‐GOE transfer of RCs from a Cyanobacteria stem lineage to the anoxygenic phototrophs in the 1.2 akinete‐constrained Model D is feasible but not guaranteed as the stem and crown ages are 2776 Ma and 2244 Ma, respectively.

### 3.4 Inferring ancestry from reaction center diversity and evolution

An additional constraint on photosystem evolution that has previously been investigated is the molecular organization of the reaction center proteins themselves. RCII subunits show heterodimeric structure arising from multiple, independent gene duplication events that occurred early in their evolution, as evidenced by maximum‐likelihood phylogenetic reconstructions (Cardona, 2015). Many evolutionary scenarios are consistent with the observed distribution of RCII across phototrophic groups, although the paraphyletic relationship between L and M types of RCII in Proteobacteria and GNS argues against RCII HGT between Cyanobacteria and either of these groups after gene duplications occurred (Cardona, 2015; Cardona et al., 2017). The evolutionary pattern of L and M types of RCII, however, is consistent with HGT of both heterodimer subunits between the stem lineages of GNS and PSB, after their divergence in a donor lineage. The phylogeny of these subunits does not constrain the lineage in which the paralog ancestor of RCII first emerged or the direction of any subsequent HGT. The structural differences between the cyanobacterial and L/M type RC proteins likely emerged after any putative HGT events, representing more derived states within their recipient lineages. Presumably, before their respective duplications, the RCII ancestors (in both cases) were homodimeric and likely much more similar to one another.

The very long branch lengths separating these RCII types and paralogs and the lack of information for root placement limit the value of directly estimating the relative or absolute timing of these events with a molecular clock approach. Regardless of the specific history of each RC subunit, structural studies support that extensive modifications have occurred within each RCII paralog lineage, especially within cyanobacterial heterodimeric paralogs as part of the evolution of the water‐evolving complex. The phylogeny of RCI proteins shows a similar evolutionary signal, although in this case, RCI complexes within GSB, Acidobacteria, and Heliobacteria are homodimeric while the cyanobacterial type is a heterodimer resulting from a gene duplication (Cardona, 2015; Cardona et al., 2017). Similar to RCII, the unrooted tree topology, limited sequence information, and long branch lengths imposed by very high evolutionary rates prevent placement of the root along the deep branches separating RCI types. As such, the organismal lineages traversed by these deep branches cannot be directly inferred nor can the direction of any putative HGT between these groups.

It remains entirely possible that the origins of each RC pre‐date the extant phototrophic diversity that can be sampled (Cardona et al., 2017), as these deep histories are neither sufficiently constrained by RC phylogenies or species tree molecular clocks. Hypotheses explaining the extant diversity of phototrophic microbial lineages as a product of these deep evolutionary processes therefore include (i) a phototrophic ancestor deep in the bacterial tree, with a pattern of RC diversification and extensive photosystem gene loss giving rise to the observed extant phototrophic diversity (Cardona et al., 2017) or (ii) an RC ancestor either within an extinct or extant phototrophic lineage and a combination of gene duplication and HGT events giving rise to the current distribution and diversity of extant phototrophic lineages. While the molecular clocks presented here cannot directly evaluate the hypothesis of vertical inheritance, or the likelihood of such extensive gene loss thereby implied, dating species tree lineages containing these photosystems can constrain the subset of hypotheses involving HGT events occurring between extant stem lineages, even if, in some cases, the HGT donor clades remain unsampled or no longer exist.

## 4 CONCLUSIONS

Using HGT‐constrained molecular clocks, we are able to obtain more precise divergence date estimates of GNS and other phototrophic taxa. These molecular clocks indicate that GNS and Cyanobacteria are older than GSB and that the transfer of FeS‐Type RCs from stem GSB to stem Cyanobacteria is less probable than the transfer of Quinone‐Type RCs from stem GNS to stem Cyanobacteria. Depending on the akinete constraint used, crown Cyanobacteria (2244 Ma; 1.2 Ga akinete‐constrained Model D) or thylakoid membrane containing Cyanobacteria (2234 Ma; 1.6 Ga akinete‐constrained Model D) most likely appear shortly after the GOE (~2.3 Ga) while crown GSB (~1.7 to 1.8 Ga; all Models A and D) emerge just after the loss of the banded iron formations from the sedimentary record ~1.8 Ga. These events represent major changes in global chemistry and, potentially, indicate expansions of crown bacterial taxa into new environmental niches. Further constraining and testing these age ranges via additional fossil calibrations and HGT events will prove essential in establishing the direction of ancient photosystem HGTs and more tightly linking the genomic and geological records.
