# Supplementary material for: Dating phototrophic microbial lineages with reticulate gene histories
Source: Geobiology. 2018 Jan 31;16(2):179–89. doi: 10.1111/gbi.12273 (PMC5873394; doi:10.1111/gbi.12273)
Supplement: Supplementary file 1 [file GBI-16-179-s001.docx]

**Supplementary Materials:**

**Dating phototropic microbial lineages with reticulate gene histories**

**Phototroph results under the best scoring ML-tree**

The best scoring ML-Tree (Supplementary Data 2) was not selected for analysis in the main text as it was in disagreement with 16S SSU Ribosomal RNA phylogenies previously reported [1,2]. The discrepancy between trees was due to differences in the placement of Sericytochromatia. The 16S SSU Ribosomal RNA places Sericytochromatia basal to the Cyanobacteria/Melainabacteria group (Supplementary Data 5). Under the best scoring ML-Tree, the non-photosynthetic lineages (Sericytochromatia and Melainabacteria) are placed sister to the oxygenic phototrophs (Cyanobacteria).

Under the best scoring ML-Tree, the date estimates for Cyanobacteria (Supplement Table 3) were only slightly older than the date estimates of the models reported in the main text. In the non-HGT constrained Model A, the change in GMS group topology did not affect the divergence date estimates of GNS and GSB. However, when the HGT constraints of Model D are applied, younger divergence date estimates of GNS are obtained (Supplement Table 3).

**Phototroph results under the prior (Guided Tree)**

Divergence date estimates were also estimated under the prior using the “-prior” option in PhyloBayes. Chronograms for these analyses are provided in Supplementary Data 12. In general, the precision of phototroph models under the prior was less than when analyses were performed using the sequence data and likelihood computations.

**Model B and C results (Guided Tree)**

The phototroph divergence date estimates of Models B and C are listed in Supplement Table 4. These results of these models indicate the at GNS are very sensitive to the HGT constraints applied. With respect to GNS, both Model B and C estimate crown and stem ages that are older than the non-HGT constrained Model A.

**Estimating the probability that a stem date estimate is older than a crown age estimate**

The probability that a stem lineage is older than a crown lineage is obtained from the posterior distributions of the stem (*s*) and crown (*c*) lineages via the following formulation.

$$P\left( s>c \right)= 1-P(c<s)$$

$$P\left( c<s \right)= \int P\left( c<x \right)ds\left( x \right)=\int F_{c}\left( x \right)s\left( x \right)dx$$

Note that *F_c_* is used to indicate the cumulative distribution function of *c*.

**Supplementary Tables:**

**Supplement Table 1:** Taxa included in this study

| Species Name | Database | Genome ID | PhyloBayes ID |
| --- | --- | --- | --- |
| *Rickettsia typhi* str. B9991CWPP | PATRIC | 1003202.3 | A73 |
| *Parvularcula bermudensis* HTCC2503 | PATRIC | 314260.8 | A74 |
| *Pelagibaca bermudensis* HTCC2601 | PATRIC | 314265.3 | A75 |
| *Rhodospirillum centenum* SW | PATRIC | 414684.5 | A76 |
| *Methylobacterium radiotolerans* JCM 2831 | PATRIC | 426355.14 | A77 |
| *Caulobacter segnis* ATCC 21756 | PATRIC | 509190.6 | A78 |
| *Candidatus Pelagibacter ubique* HTCC1062 | PATRIC | 335992.9 | A80 |
| *Pedobacter kyungheensis* KACC 16221 | PATRIC | 1069985.4 | B12 |
| *Blattabacterium* sp. *(Cryptocercus punctulatus)* str. Cpu | PATRIC | 1075399.3 | B13 |
| *Formosa* sp. AK20 | PATRIC | 1137281.3 | B14 |
| *Mangrovimonas yunxiaonensis*str. LY01 | PATRIC | 1197477.3 | B15 |
| *Cellulophaga geojensis* KL-A | PATRIC | 1328323.3 | B16 |
| *Cardinium endosymbiont* cBtQ1 of Bemisia tabaci | PATRIC | 1354314.3 | B17 |
| *Elizabethkingia meningoseptica* 502 | PATRIC | 1355388.3 | B18 |
| *Rikenellaceae bacterium* M3 | PATRIC | 1433126.3 | B19 |
| *Flavobacterium aquatile* LMG 4008 | PATRIC | 1453498.8 | B20 |
| *Schleiferia thermophila str Yellowstone* | PATRIC | 1453500.3 | B21 |
| *Chryseobacterium antarcticum* | PATRIC | 266748.6 | B22 |
| *Capnocytophaga canimorsus* str. CcD38 | PATRIC | 28188.6 | B23 |
| *Robiginitalea biformata* HTCC2501 | PATRIC | 313596.4 | B24 |
| *Porphyromonas canoris* COT-108_OH1224 | PATRIC | 36875.4 | B25 |
| *Kordia algicida* OT-1 | PATRIC | 391587.3 | B26 |
| *Epilithonimonas lactis* str. LMG 24401 | PATRIC | 421072.8 | B27 |
| *Chryseobacterium luteum*str. DSM 18605 | PATRIC | 421531.5 | B28 |
| *Parabacteroides distasonis* ATCC 8503 | PATRIC | 435591.13 | B29 |
| *Bacteroides fragilis* 3_1_12 | PATRIC | 457424.5 | B30 |
| *Spirosoma linguale* DSM 74 | PATRIC | 504472.7 | B31 |
| *Nonlabens dokdonensis* DSW-6 | PATRIC | 592029.3 | B32 |
| *Marivirga tractuosa* DSM 4126 | PATRIC | 643867.3 | B33 |
| *Paludibacter propionicigenes* WB4 | PATRIC | 694427.4 | B34 |
| *Odoribacter splanchnicus* DSM 220712 | PATRIC | 709991.3 | B35 |
| *Paraprevotella clara* YIT 11840 | PATRIC | 762968.3 | B36 |
| *Weeksella virosa* DSM 16922 | PATRIC | 865938.3 | B37 |
| *Ornithobacterium rhinotracheale* DSM 15997 | PATRIC | 867902.3 | B38 |
| *Niabella soli* DSM 19437 | PATRIC | 929713.3 | B39 |
| *Prevotella nigrescens* ATCC 33563 | PATRIC | 997352.4 | B40 |
| *Fluviicola taffensis* DSM 16823 | PATRIC | 755732.3 | B43 |
| *Owenweeksia hongkongensis* DSM 17368 | PATRIC | 926562.3 | B44 |
| *Blattabacterium* sp. *(Blaberus giganteus)* | PATRIC | 1186051.3 | B79 |
| *Nitrolancetus hollandicus* Lb | PATRIC | 1129897.3 | Cf63 |
| *Dehalococcoides mccartyi* CG1 | PATRIC | 1432059.3 | Cf64 |
| *Thermomicrobium roseum* DSM 5159 | PATRIC | 309801.4 | Cf65 |
| *Chloroflexus aurantiacus* J-10-fl | PATRIC | 324602.8 | Cf66 |
| *Chloroflexus aggregans* DSM 9485 | PATRIC | 326427.4 | Cf67 |
| *Sphaerobacter thermophilus* DSM 20745 | PATRIC | 479434.6 | Cf68 |
| *Ktedonobacter racemifer* DSM 44963 | PATRIC | 485913.3 | Cf69 |
| *Dehalogenimonas lykanthroporepellens* BL-DC-9 | PATRIC | 552811.9 | Cf70 |
| *Caldilinea aerophila* DSM 14535 = NBRC 104270 | PATRIC | 926550.5 | Cf71 |
| *Anaerolinea thermophila* UNI-1 | PATRIC | 926569.3 | Cf72 |
| *Roseiflexus* sp. RS-1 | PATRIC | 357808.5 | Cf81 |
| *Roseiflexus castenholzii* DSM 13941 | PATRIC | 383372.6 | Cf82 |
| *Oscillochlorois trichoides* | NCBI | GCF_000152145.1_ASM15214v1 | Cf83 |
| *Chlorobium tepidum* TLS | PATRIC | 194439.7 | Chl0 |
| *Chlorobium phaeobacteroides* BS1 | PATRIC | 331678.5 | Chl1 |
| *Chlorobium ferrooxidan*DSM 13031 | PATRIC | 377431.3 | Chl10 |
| *Chlorobium chlorochromatii* CaD3 | PATRIC | 340177.11 | Chl11 |
| *Chlorobium phaeovibrioides* DSM 265 | PATRIC | 290318.6 | Chl2 |
| *Chlorobium luteolum* DSM 273 | PATRIC | 319225.5 | Chl3 |
| *Chlorobium phaeobacteroides* DSM 266 | PATRIC | 290317.9 | Chl4 |
| *Chlorobaculum parvum* NCIB 8327 | PATRIC | 517417.5 | Chl5 |
| *Chlorobium limicola* DSM 245 | PATRIC | 290315.5 | Chl6 |
| *Pelodictyon phaeoclathratiforme* BU-1 | PATRIC | 324925.5 | Chl7 |
| *Chloroherpeton thalassium* ATCC 35110 | PATRIC | 517418.5 | Chl8 |
| *Prosthecochloris aestuarii* DSM 271 | PATRIC | 290512.6 | Chl9 |
| *Leptolyngbya* sp. PCC 7375 | PATRIC | 102129.3 | Cyn45 |
| *Nostoc* sp. PCC 7120 | PATRIC | 103690.1 | Cyn46 |
| *Stanieria cyanosphaera* PCC 7437 | PATRIC | 111780.3 | Cyn47 |
| *Fischerella* sp. JSC-11 | NCBI | GCF_000231365.1_ASM23136v1 | Cyn48 |
| *Geitlerinema* sp. PCC 7407 | PATRIC | 1173025.3 | Cyn49 |
| *Coleofasciculus chthonoplastes* PCC 7420 | PATRIC | 118168.3 | Cyn50 |
| *Prochlorococcus marinus* subsp. marinus str. CCMP1375 | PATRIC | 167539.5 | Cyn51 |
| *Thermosynechococcus elongatus* BP-1 | PATRIC | 197221.4 | Cyn52 |
| *Trichodesmium erythraeum* IMS101 | PATRIC | 203124.6 | Cyn53 |
| *Gloeobacter violaceus* PCC 7421 | PATRIC | 251221.4 | Cyn54 |
| *Chroococcidiopsis thermalis* PCC 7203 | PATRIC | 251229.3 | Cyn55 |
| *Synechococcus elongatus* PCC 6301 | PATRIC | 269084.6 | Cyn56 |
| *Cyanobium gracile* PCC 6307 | PATRIC | 292564.3 | Cyn57 |
| *Prochlorothrix hollandica*PCC 9006 = CALU 1027 | NCBI | GCF_000332315.1_ASM33231v1 | Cyn58 |
| *Rivularia* sp. PCC 7116 | PATRIC | 373994.3 | Cyn59 |
| *Cyanothece* sp. PCC 7822 | PATRIC | 497965.6 | Cyn60 |
| *Arthrospira platensis* NIES-39 | PATRIC | 696747.3 | Cyn61 |
| *Pseudanabaena* sp. PCC 7367 | PATRIC | 82654.3 | Cyn62 |
| *Melioribacter roseus* P3M-2 | PATRIC | 1191523.3 | Ig41 |
| *Ignavibacterium album* JCM 16511 | PATRIC | 945713.3 | Ig42 |
| *Wolbachia* endosymbiont of *Drosophila simulans* wNo | NCBI | GCA_000376585.1_ASM37658v1 | p1236908 |
| *Anaplasma phagocytophilum* str. Webster | NCBI | GCA_000964685.1_ASM96468v1 | p1359162 |
| *Magnetococcus marinus* MC-1 | NCBI | GCA_000014865.1_ASM1486v1 | p156889 |
| *Ehrlichia canis* str. Jake | NCBI | GCA_000012565.1_ASM1256v1 | p269484 |
| *Obscuribacter phosphatis* (Mle1_12) | IMG | 2556921048 | M_p26868 |
| *Gastranaerophilaceae* Zag_1 (Zagget bin 1) | IMG | 2523533517 | p2523533517 |
| *Gastranaerophilaceae* Zag_111 (Zagget_111_MP) | IMG | 2531839741 | M_p19866 |
| *Gastranaerophilus phascolarctosicola* (Zagget bin 221) | IMG | 2523533519 | M_p13725 |
| *Sericytochromatia* (*Serocytochromales* representative)  Obtained from Coal Bed ML635J-21 | R. Soo | CBMW_12 | U_71173 |
| *Sericytochromatia* (*Serocytochromales* representative)  Obtained from Rifle Acetate Amendment | R. Soo | RAAC_196 | U_67084 |
| *Sericytochromatia* (*GL2-53* representative)  Obtained from Photoreactor Bin 72 | R. Soo | LSPB_72 | U_41057 |

**Supplement Table 2:** Proteins used in the concatenated alignment and the order in which they were aligned

| Protein Name | Alignment Length |
| --- | --- |
| Ribosomal Protein L1 | 259 |
| Ribosomal Protein L2 | 297 |
| Ribosomal Protein L3 | 383 |
| Ribosomal Protein L4 | 295 |
| Ribosomal Protein L5 | 220 |
| Ribosomal Protein L6 | 220 |
| Ribosomal Protein L10 | 238 |
| Ribosomal Protein L13 | 196 |
| Ribosomal Protein L14 | 135 |
| Ribosomal Protein L15 | 215 |
| Ribosomal Protein L18 | 191 |
| Ribosomal Protein L22 | 187 |
| Ribosomal Protein L23 | 193 |
| Ribosomal Protein L24 | 209 |
| Ribosomal Protein L29 | 140 |
| Ribosomal Protein S2 | 436 |
| Ribosomal Protein S3 | 366 |
| Ribosomal Protein S4 | 252 |
| Ribosomal Protein S5 | 301 |
| Ribosomal Protein S7 | 172 |
| Ribosomal Protein S8 | 144 |
| Ribosomal Protein S9 | 174 |
| Ribosomal Protein S10 | 159 |
| Ribosomal Protein S11 | 160 |
| Ribosomal Protein S12 | 177 |
| Ribosomal Protein S13 | 135 |
| Ribosomal Protein S14 | 113 |
| Ribosomal Protein S15 | 136 |
| Ribosomal Protein S17 | 150 |
| Ribosomal Protein S19 | 105 |

**Supplement Table 3:** Phototroph Results under the best scoring ML-Tree

|  | **Node**  **ID** | **1.2 Ga Akinete Constraint** | | **1.6 Ga Akinete Constraint** | |
| --- | --- | --- | --- | --- | --- |
|  |  | **Model A** | **Model D*** | **Model A** | **Model D*** |
| **Root Age** | **1** | **3496 Ma**  (3277 – 3806 Ma) | **3538 Ma**  (3239 – 3839 Ma) | **3628 Ma**  (3397 – 3910 Ma) | **3674 Ma**  (3461 – 3906 Ma) |
| **Stem Cyanobacteria** | **2** | **2920 Ma**  (2661 – 3212 Ma) | **3007 Ma**  (2704 – 3253 Ma) | **3254 Ma**  (2942 – 3555 Ma) | **3285 Ma**  (3008 – 3513 Ma) |
| **Crown Cyanobacteria** | **3** | **2451 Ma**  (2142 – 2687 Ma) | **2429 Ma**  (2236 – 2687 Ma) | **2813 Ma**  (2535 – 3103 Ma) | **2855 Ma**  (2565 –3107 Ma) |
| **Crown Cyanobacteria**  **Excluding *Gloeobacter*** | **4** | **2086 Ma**  (1835 – 2314 Ma) | **2104 Ma**  (1933 – 2350 Ma) | **2470 Ma**  (2258 – 2770 Ma) | **2505 Ma**  (2330 – 2746 Ma) |
| **Stem GNS** | **5** | **1977 Ma**  (1535 – 2498 Ma) | **2553 Ma**  (2339 – 2875 Ma) | **2160 Ma**  (1600– 2626 Ma) | **2493 Ma**  (2193 – 2916 Ma) |
| **Crown GNS** | **6** | **1063 Ma**  (729 – 1543 Ma) | **1805 Ma**  (1658 – 2105 Ma) | **1145 Ma**  (721 – 1648 Ma) | **1845 Ma**  (1667 – 2162 Ma) |
| **Stem GSB** | **7** | **2536 Ma**  (2219 – 2901 Ma) | **2435 Ma**  (2174 – 2810 Ma) | **2527 Ma**  (2175 – 2846 Ma) | **2452 Ma**  (2134 – 2748 Ma) |
| **Crown GSB** | **8** | **1791 Ma**  (1640 – 2071 Ma) | **1725 Ma**  (1646 – 1944 Ma) | **1822 Ma**  (1641 – 2152 Ma) | **1715 Ma**  (1640 – 1894 Ma) |

*SahH HGT constraint applied to crown Cyanobacteria instead of crown Cyanobacteria/Melainabacteria group.

**Supplement Table 4:** Model B and C Results (Guided Tree)

|  | **Node**  **ID** | **1.2 Ga Akinete Constraint** | | **1.6 Ga Akinete Constraint** | |
| --- | --- | --- | --- | --- | --- |
|  |  | **Model B** | **Model C** | **Model B** | **Model C** |
| **Root Age** | **1** | **3595 Ma**  (3410 – 3884 Ma) | **3663 Ma**  (3378 – 3946 Ma) | **3713 Ma**  (3569 – 3977 Ma) | **3716 Ma**  (3440 – 3994 Ma) |
| **Stem Cyanobacteria** | **2** | **2719 Ma**  (2322 – 3047 Ma) | **2958 Ma**  (2631 – 3300 Ma) | **2856 Ma**  (2724 – 3275 Ma) | **3162 Ma**  (2852 – 3475 Ma) |
| **Crown Cyanobacteria** | **3** | **2224 Ma**  (1986 – 2544 Ma) | **2393 Ma**  (2072 – 2685 Ma) | **2519 Ma**  (2382 – 2880 Ma) | **2676 Ma**  (2413 – 2952 Ma) |
| **Crown Cyanobacteria**  **Excluding *Gloeobacter*** | **4** | **1901 Ma**  (1692 – 2140 Ma) | **2011 Ma**  (1773 – 2280 Ma) | **2249 Ma**  (2082 – 2470 Ma) | **2342 Ma**  (2126 – 2575 Ma) |
| **Stem GNS** | **5** | **2748 Ma**  (2488 – 3110 Ma) | **2444 Ma**  (2100 – 2841 Ma) | **2908 Ma**  (2759– 3312 Ma) | **2536 Ma**  (2156 – 2972 Ma) |
| **Crown GNS** | **6** | **1818 Ma**  (1653 – 2438 Ma) | **1832 Ma**  (1646 – 2117 Ma) | **2006 Ma**  (1704 – 2438 Ma) | **1871 Ma**  (1643 – 2218 Ma) |
| **Stem GSB** | **7** | **2557 Ma**  (2223 – 2853 Ma) | **2558 Ma**  (2198 – 2916 Ma) | **2714 Ma**  (2423 – 2902 Ma) | **2585 Ma**  (2236 – 2957 Ma) |
| **Crown GSB** | **8** | **1763 Ma**  (1645 – 2161 Ma) | **1704 Ma**  (1640 – 1893 Ma) | **1805 Ma**  (1660 – 2173 Ma) | **1724 Ma**  (1640 – 1935 Ma) |

**Supplement Table 5:** Phototroph model with no internal calibrations (Guided Tree)

|  | **Node ID** | **No Internal Calibrations** |
| --- | --- | --- |
| **Root Age** | **1** | **3899 Ma**  (3554 – 4239 Ma) |
| **Stem Cyanobacteria** | **2** | **2892 Ma**  (2496 – 3279 Ma) |
| **Crown Cyanobacteria** | **3** | **2172 Ma**  (1841 – 2569 Ma) |
| **Crown Cyanobacteria**  **Excluding *Gloeobacter*** | **4** | **1696 Ma**  (1424 – 2053 Ma) |
| **Stem GNS** | **5** | **2250 Ma**  (1754 – 2799 Ma) |
| **Crown GNS** | **6** | **1085 Ma**  (708 – 1579 Ma) |
| **Stem GSB** | **7** | **2844 Ma**  (2271 – 3269 Ma) |
| **Crown GSB** | **8** | **1694 Ma**  (1222 – 2140 Ma) |

**Supplement Figure 1:** Tip labels for the phylogenetic tree of Figure 1

**Supplementary Data:**

All supplementary data is provided in the text file “Geobiology_Supplement_Data.txt”. To split the data file into the individual supplementary data files listed below, execute the python script “split_supplement_master_file.py.txt”

**Supplementary Data 1:** Concatenated Ribosomal Alignment

**Supplementary Data 2:** Best Scoring Ribosomal Tree

**Supplementary Data 3:** Guide tree used as input for RAxML

**Supplementary Data 4:** 30 ribosomal tree used in this study

**Supplementary Data 5:** 16S SSU Ribsomal RNA tree

**Supplementary Data 6:** *datedist and summary files for PhyloBayes runs

**Supplementary Data 7:** *Gloeobacter* Outgroup ML Tree

**Supplementary Data 8:** Alphaproteobacteria Outgroup ML Tree

**Supplementary Data 9:** Calibration files for PhyloBayes

**Supplementary Data 10:** BchH ML Tree and alignment

**Supplementary Data 11:** SahH ML Tree and alignment

**Supplementary Data 12:** Chronograms for posterior estimates obtained under the prior
